# Retinal, Vascular, and Choroidal Remodeling After Non-Reperfused Central Retinal Artery Occlusion: A Longitudinal OCT Study

**DOI:** 10.3390/diagnostics16142159

**Published:** 2026-07-10

**Authors:** Tahsin Akçaoğlu, Gökhan Pekel, Emine Seker Un

**Affiliations:** 1 Department of Ophthalmology, Denizli State Hospital, Denizli 20010, Türkiye; 2 Department of Ophthalmology, Faculty of Medicine, Pamukkale University, Denizli 20160, Türkiye

**Keywords:** central retinal artery occlusion, retinal vessel diameter, macular segmentation, optical coherence tomography, non-reperfused CRAO, longitudinal imaging

## Abstract

**Background**: This study aimed to evaluate longitudinal changes in retinal layers, retinal vessel diameters, and choroidal thickness using optical coherence tomography (OCT) in patients with central retinal artery occlusion (CRAO). **Methods**: This retrospective study included 35 patients (70 eyes) diagnosed with unilateral CRAO between 1 January 2020 and 5 January 2024. Unaffected fellow eyes served as internal controls. OCT-based macular segmentation, retinal arterial and venous diameter measurements, and subfoveal and peripapillary choroidal thickness were assessed in the acute phase (<24 h) and at 1-month and 6-month follow-up visits. Subgroup analyses compared patients who received hyperbaric oxygen therapy (HBOT) with those who did not. Statistical analyses included *t*-tests, Mann–Whitney U, Wilcoxon signed-rank, and chi-square tests. **Results**: Retinal arterial and venous diameters showed significant narrowing during follow-up (arteries: 77.91  ±  11.77 µm to 70.74  ±  15.12 µm; veins: 130.94  ±  22.54 µm to 118.57  ±  20.61 µm; both *p*  <  0.05). Macular segmentation demonstrated marked thinning of the inner retinal layers, most prominently in the retinal nerve fiber layer and ganglion cell layer (40.09 ± 26.86 to 12.51 ± 4.99 µm and 61.20 ± 25.83 to 24.26 ± 12.11 µm, respectively; both *p* = 0.001). Subfoveal choroidal thickness progressively decreased over time (from 206.40 ± 34.91 µm to 178.86 ± 28.28 µm, *p* = 0.009). Peripapillary choroidal thickness increased across all quadrants at 6 months, most consistently in the inferior quadrant (*p* = 0.001). No significant differences in retinal or choroidal parameters were observed between HBOT-treated and non-treated patients. All cases exhibited persistent vascular occlusion without reperfusion. **Conclusions**: CRAO is characterized by progressive inner retinal atrophy, retinal vessel narrowing, and distinct temporal changes in choroidal morphology. The relative preservation and subsequent increase in peripapillary choroidal thickness during follow-up suggest dynamic choroidal involvement despite persistent occlusion. OCT-based macular segmentation and retinal vessel analysis provide objective biomarkers for longitudinal structural assessment in CRAO. By jointly quantifying retinal layer, retinal vascular, and choroidal remodeling, these findings may also provide reference data for future studies of structural evolution in non-reperfused CRAO.

## 1. Introduction

Central retinal artery occlusion (CRAO) is an ophthalmic emergency that typically presents with sudden, painless, unilateral vision loss caused by an abrupt interruption of retinal arterial blood flow, resulting in ischemic injury analogous to cerebral stroke [1]. Without timely intervention, retinal ischemia often leads to permanent structural damage and profound visual impairment. Among CRAO subtypes, emboli originating from atherosclerotic plaques in the internal carotid artery represent the most common etiology [2]. Other causes include in situ thrombosis related to atherosclerosis, collagen vascular diseases, inflammatory conditions, and hypercoagulable states [3].

The inner retinal layers including the retinal nerve fiber layer, ganglion cell layer, and inner plexiform layer are primarily supplied by the central retinal artery and its branches [4,5]. Acute arterial occlusion results in retinal ischemia, initially manifesting as intracellular edema and increased thickness of the inner retinal layers, followed by progressive atrophy if perfusion is not restored [6,7]. Characteristic funduscopic findings include retinal arterial attenuation, optic disc edema, diffuse retinal whitening, and the classic cherry-red spot at the macula [6,7].

Optical coherence tomography (OCT) has emerged as a critical non-invasive imaging modality for assessing structural alterations in CRAO, providing detailed, quantitative information on retinal morphology. Early OCT-based studies in CRAO primarily described global retinal and macular thickness changes and highlighted characteristic inner retinal involvement, particularly during the acute phase of the disease [8,9]. Subsequent investigations expanded this approach by performing more detailed analyses of individual retinal layers using OCT-based segmentation techniques, demonstrating layer-specific structural alterations associated with ischemic injury [9].

In parallel, other studies have explored vascular-related parameters or choroidal involvement in CRAO, reporting changes in retinal vessel characteristics or subfoveal choroidal thickness and their associations with disease severity and visual prognosis [10,11]. However, these studies have generally evaluated retinal layers, retinal vasculature, or choroidal parameters separately and often included heterogeneous cohorts comprising partially or completely reperfused cases. As a result, the integrated and longitudinal structural evolution of CRAO-related retinal and choroidal alterations in persistently non-reperfused eyes remains incompletely understood.

Consequently, the longitudinal morphological evolution of CRAO without reperfusion—when retinal layer integrity, retinal vessel diameters, and choroidal thickness are evaluated together within a single analytical framework—has not been comprehensively characterized. Therefore, the primary aim of this study was to quantitatively assess longitudinal changes in retinal layers, retinal vessel diameters, and choroidal thickness in patients with non-reperfused CRAO using OCT-based macular segmentation and vascular measurements. By generating an integrated set of objective quantitative measurements across retinal, retinal vascular, and choroidal compartments, this study also sought to provide a structural reference framework for future investigations of CRAO-related remodeling. In addition, as a secondary and exploratory analysis, structural parameters were compared between patients who received adjunctive hyperbaric oxygen therapy (HBOT) and those who did not, to further characterize potential treatment-associated differences within this non-reperfused cohort [11].

## 2. Methods

This retrospective study was conducted following approval from the institutional ethics committee and in accordance with the tenets of the Declaration of Helsinki. The study was approved by the Non-Interventional Clinical Research Ethics Committee of Pamukkale University (approval number: E.474221; date: 10 January 2024). Medical records of patients diagnosed with unilateral central retinal artery occlusion (CRAO) who presented to the emergency department and were subsequently consulted, followed, and treated by our ophthalmology team between 1 January 2020 and 5 January 2024 were reviewed. Demographic characteristics and disease-related clinical data were obtained from patient records. At baseline, best-corrected visual acuity (BCVA), intraocular pressure (IOP), anterior and posterior segment examination findings, fundus photographs, and optical coherence tomography images were recorded. These assessments were repeated at follow-up visits. Visual acuity was initially measured using the Snellen chart and converted to logarithm of the minimum angle of resolution (logMAR) for statistical analysis. In patients with very low vision, counting fingers measurements at standardized distances were also recorded to avoid overlooking minor visual differences. A total of 35 patients (70 eyes) were included, with the unaffected fellow eyes serving as internal controls.

Inclusion criteria consisted of patients diagnosed with unilateral CRAO who presented within 24 h of symptom onset (acute phase) and demonstrated persistent vascular occlusion without reperfusion throughout follow-up. The fellow eye was required to have no ocular pathology and normal OCT findings.

Exclusion criteria included presentation during the atrophic phase, presence of an intact cilioretinal artery or isolated cilioretinal artery occlusion, retinal pathology in the control eye, bilateral retinal artery occlusion, retinal artery occlusion secondary to trauma, intraocular surgery within the previous three months, combined retinal artery and vein occlusion, patients referred after treatment had already been initiated elsewhere, and eyes demonstrating spontaneous or treatment-associated reperfusion during follow-up, in order to ensure a homogeneous non-reperfused CRAO cohort.

For ethical reasons, no patient was left untreated. All patients received standard acute management for central retinal artery occlusion, including ocular massage and intraocular pressure-lowering therapy with a fixed combination of dorzolamide and timolol [12,13]. Hyperbaric oxygen therapy was administered to clinically eligible patients according to a standardized institutional protocol. Each HBOT session consisted of two 45 min exposures per day (total of 90 min), delivered at 2.4 atmospheres absolute (ATA), for a total of 10 sessions [10]. Among the study population, 25 patients received HBOT, while 10 patients did not. Reasons for not receiving HBOT included delayed presentation beyond the optimal treatment window, medical contraindications, or patient refusal. HBOT exposure status was documented, and subgroup analyses were performed within the non-reperfused cohort. This study was not designed to evaluate the efficacy of HBOT but rather to characterize morphological changes in CRAO cases without reperfusion.

**Figure 1:**
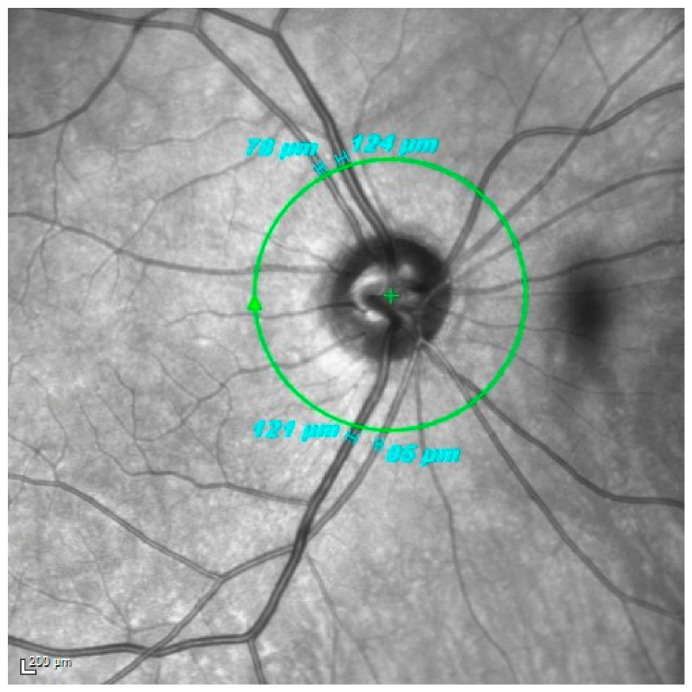
Retinal vessel diameters were measured using the **optical coherence tomography (OCT)** software’s manual caliper tool at one optic disc diameter from the disc margin, with calipers placed on the outer vessel borders after 400% magnification.

All retinal imaging was performed using a spectral-domain OCT device (Heidelberg Spectralis; Heidelberg Engineering GmbH, Heidelberg, Germany). Retinal artery and vein diameters were evaluated at baseline and at the final follow-up visit. Optic disc-centered OCT scans were obtained using the device’s automatic calibration system (Spectralis software version 5.8). The superior and inferior temporal arterioles and venules located one optic disc diameter from the disc margin were selected. Vessel diameters were measured manually using the built-in calipers placed on the outer vessel boundaries at 400% magnification, and the mean of the superior and inferior measurements was calculated (Figure 1). All OCT examinations were performed at similar times of day to minimize the potential effect of diurnal variation. In addition, all scans were acquired using the same OCT device and the same software version throughout the study.

**Figure 2:**
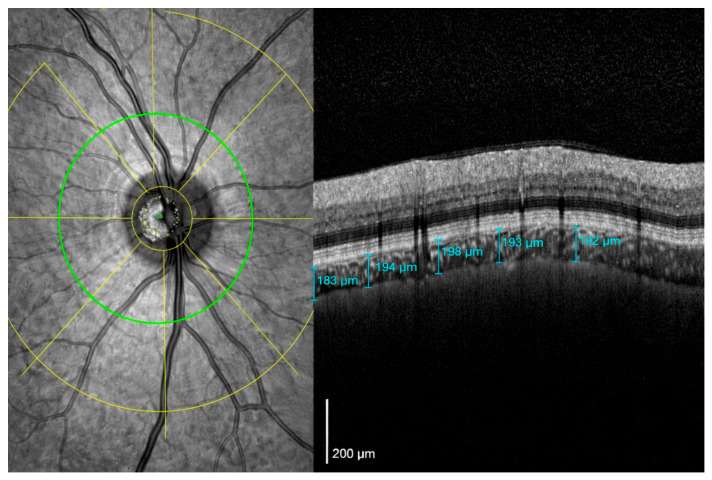
Representative peripapillary choroidal thickness measurement on enhanced depth imaging (EDI) OCT. Choroidal thickness was defined as the vertical distance between the outer border of the retinal pigment epithelium–Bruch’s membrane complex and the choroid–sclera junction, measured at multiple peripapillary points. Scale bar = 200 μm.

Peripapillary choroidal thickness was measured in the optic disc imaging mode from the nasal, temporal, superior, and inferior quadrants. Choroidal thickness was defined as the distance between the outer border of the hyperreflective retinal pigment epithelium and the inner surface of the sclera (Figure 2).

**Figure 3:**
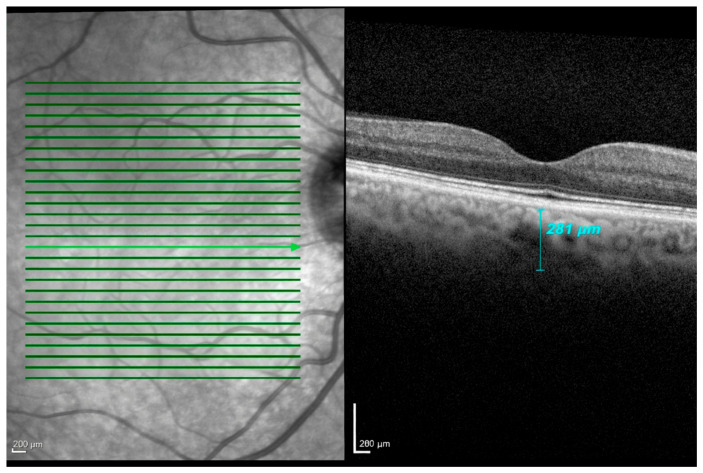
Subfoveal choroidal thickness was defined as the distance between the outer border of the retinal pigment epithelium and the choroid–sclera interface at the fovea, measured using macular scans.

Subfoveal choroidal thickness was measured in the macular scan mode at the foveal center using the same anatomical boundaries (Figure 3).

**Figure 4:**
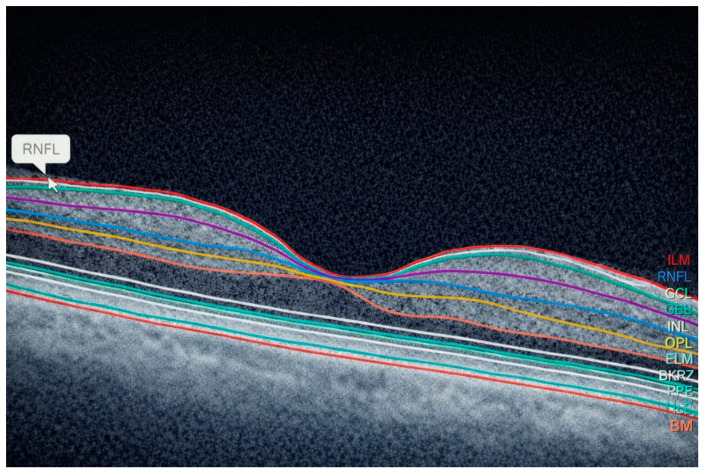
Macular layer segmentation on **spectral-domain optical coherence tomography (SD-OCT)**. Representative SD-OCT image showing automated macular layer segmentation with color-coded retinal layers.

Macular segmentation was performed on macular OCT scans, including measurements of the retinal nerve fiber layer (RNFL), ganglion cell layer (GCL), inner plexiform layer (IPL), inner nuclear layer (INL), outer plexiform layer (OPL), outer nuclear layer (ONL), and photoreceptor layer. Initial segmentation was performed using the device’s automated software (Figure 4). Manual correction was applied only to adjust segmentation boundaries in regions affected by edema or structural irregularities and was not used to obtain independent manual thickness measurements. Layer thickness was defined as the distance between the outer boundary of each layer and the outer boundary of the adjacent layer. Quantitative retinal layer thickness values were derived from automated macular OCT segmentation and expressed as mean thickness measurements, rather than single-point or ETDRS grid-based regional values.

**Figure 5:**
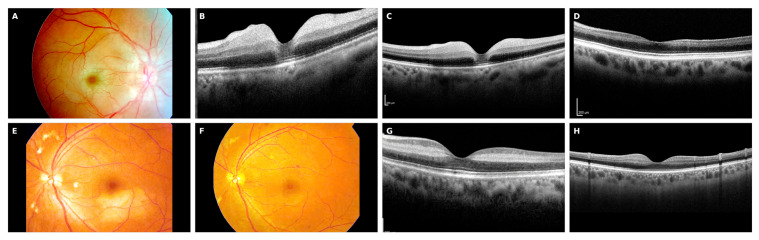
Representative fundus and OCT findings used for reperfusion classification in CRAO. Panels (**A**–**D**) show a non-reperfused CRAO case included in the study. Fundus photography demonstrates ischemic retinal whitening and vascular attenuation (**A**). Acute OCT shows inner retinal hyperreflectivity and thickening (**B**), followed by persistent structural ischemic changes at 1 month (**C**) and chronic inner retinal thinning/atrophy at 6 months (**D**). Panels (**E**–**H**) show a reperfused CRAO case excluded from the analysis. Acute fundus photography before reperfusion is shown in panel (**E**), while follow-up fundus photography demonstrates marked reduction in retinal whitening and improvement in retinal appearance compatible with reperfusion (**F**). Acute OCT shows inner retinal hyperreflectivity and thickening (**G**), whereas follow-up OCT shows partial resolution of acute ischemic edema (**H**).

Reperfusion status was assessed retrospectively using serial dilated fundus examination, color fundus photography, macular spectral-domain OCT, and fluorescein angiography when clinically available or indicated. Persistent occlusion/non-reperfusion was defined as the absence of documented clinical or angiographic reperfusion during follow-up. Imaging findings supporting persistent occlusion included persistent retinal vascular attenuation on fundus examination and the expected OCT evolution of ischemic CRAO, characterized by acute inner retinal hyperreflectivity and thickening followed by progressive inner retinal thinning and atrophy during follow-up. Eyes showing clear evidence of reperfusion at any time point, including marked reduction in retinal whitening, improvement in vascular appearance, angiographic evidence of restored retinal perfusion when available, and partial or complete resolution of acute inner retinal edema on OCT, were classified as reperfused and excluded from the analysis. Representative fundus and OCT examples of non-reperfused and reperfused cases are shown in Figure 5.

Statistical analyses were performed using IBM SPSS Statistics version 25.0 (IBM Corp., Armonk, NY, USA). Data distribution was assessed using the Kolmogorov–Smirnov test. Comparisons between groups were conducted using Student’s *t*-test for normally distributed variables and the Mann–Whitney U test for non-normally distributed variables. Within-group comparisons were performed using the paired samples *t*-test or the Wilcoxon signed-rank test, as appropriate. Categorical variables were analyzed using the chi-square test. A *p*-value < 0.05 was considered statistically significant. To assess the reliability of the manual measurements, retinal arterial and venous diameters, subfoveal choroidal thickness, and peripapillary choroidal thickness in all four quadrants were re-measured in a randomly selected subset of 20 eyes. Intraobserver reliability was assessed by the same observer on two occasions 6 days apart, masked to the initial values, and interobserver reliability by a second independent masked observer. Intraclass correlation coefficients (ICC; two-way random-effects model, absolute agreement, single measures) with 95% confidence intervals and within-subject coefficients of variation (CV) were calculated, with the acute- and chronic-phase measurements pooled. ICC values were interpreted as poor (<0.50), moderate (0.50–0.75), good (0.75–0.90), or excellent (>0.90). To account for the repeated-measures structure and the affected-versus-fellow-eye comparison, longitudinal changes were additionally analyzed using linear mixed-effects models with patient identity as a random intercept, adjusted for age, sex, diabetes mellitus, hypertension, and cardiovascular disease; an eye × visit interaction term compared the affected and fellow eyes. Axial length and refractive error were not available and were not included. These analyses are summarized in the Section 3.

## 3. Results

The patients’ ages ranged from 65 to 77 years, with a mean age of 70.03 ± 12.48 years and a median age of 71 years. There was no statistically significant age difference between female and male patients (*p* > 0.05). Measurements were performed during the acute phase and at the 1- and 6-month follow-up visits. Intraocular pressure remained stable throughout the follow-up period. During the acute phase, IOP ranged from 10 to 15 mmHg, with a mean value of 12.86 ± 3.32 mmHg. At the 1-month follow-up, IOP ranged from 9 to 16 mmHg (mean: 12.91 ± 3.19 mmHg), and at 6 months ranged from 10 to 17 mmHg (mean: 12.9 ± 3.17 mmHg). No statistically significant difference in intraocular pressure was observed across follow-up visits (*p* > 0.05). Among the study population, diabetes mellitus was present in 40% of patients, hypertension in 74.3%, and cardiovascular disease in 42.9%, with hypertension being the most common systemic comorbidity. At baseline, visual acuity was recorded as counting fingers or worse (logMAR 3.10) in 23 patients, counting fingers at 1 m (logMAR 1.80) in 8 patients, counting fingers at 2 m (logMAR 1.51) in 3 patients, and counting fingers at 3 m (logMAR 1.30) in 1 patient. At the 1-month follow-up, 23 patients continued to have visual acuity at the counting fingers level or worse (logMAR 3.10). At the final follow-up visit, 51.4% of patients still exhibited visual acuity at the counting fingers level (logMAR 3.10). No patients developed no light perception during the study period. No statistically significant difference in visual acuity was observed between patients who received hyperbaric oxygen therapy and those who did not (*p* > 0.05). Visual acuity in the fellow (control) eyes was within normal limits at baseline and remained stable throughout the follow-up period. No clinically meaningful change in visual acuity was observed in the control eyes during follow-up.

Retinal artery diameter showed a significant reduction during follow-up, decreasing from 77.91 ± 11.77 µm at baseline to 75.98 ± 12.19 µm at 1 month and 70.74 ± 15.12 µm at the final follow-up (*p* = 0.0186). Similarly, retinal vein diameter gradually decreased over time, from 130.94 ± 22.54 µm at baseline to 126.58 ± 20.67 µm at 1 month and 118.57 ± 20.61 µm at the final visit (*p* < 0.05). Subgroup analyses demonstrated similar degrees of arterial and venous narrowing among patients according to hyperbaric oxygen therapy exposure, with no statistically significant differences between subgroups (Table 1).

Macular segmentation analysis revealed significant atrophic changes and thinning predominantly in the inner retinal layers, including the retinal nerve fiber layer (RNFL), ganglion cell layer (GCL), inner plexiform layer (IPL), and inner nuclear layer (INL) at the final follow-up. Among these layers, the RNFL demonstrated the greatest initial thickening, followed by the most pronounced atrophy over time, whereas the photoreceptor layer was the least affected (Table 2).

Subgroup analysis according to hyperbaric oxygen therapy exposure revealed no statistically significant differences in macular layer thickness changes between the HBOT and non-HBOT subgroups (Table 3).

Although a more pronounced decrease in retinal layer thicknesses was observed in the HBOT group, particularly from baseline to final follow-up, these changes did not reach statistical significance and should be interpreted descriptively as longitudinal morphological changes rather than treatment-specific effects.

Subfoveal choroidal thickness in the affected eyes showed a significant decrease at the final follow-up compared to baseline. The baseline subfoveal choroidal thickness was 206.40 ± 34.91 μm, which decreased to 178.86 ± 28.28 μm at the final visit (*p* = 0.009). At the 1-month follow-up, the mean subfoveal choroidal thickness was 201.38 ± 27.87 μm. Subfoveal choroidal thickness decreased over time during follow-up. In the control group, subfoveal choroidal thickness in the healthy eyes was 202.63 ± 35.23 µm at baseline and 207.51 ± 39.83 µm at the final follow-up, with no significant difference between measurements (*p* = 0.316). Peripapillary choroidal thickness showed a mild increase at the 1-month follow-up and a significant increase at the 6-month visit compared to baseline. The increase was observed across all quadrants, with the nasal quadrant being the thickest and the inferior quadrant the thinnest. While all other measured parameters demonstrated thinning over time, peripapillary choroidal thickness was the only parameter that showed an increase. Peripapillary choroidal thickness measurements are presented in Table 4.

Measurement reliability was excellent for all manually measured parameters. Intraobserver intraclass correlation coefficients (ICCs) ranged from 0.98 to 1.00 and interobserver ICCs from 0.97 to 1.00, with all lower 95% confidence limits ≥ 0.95. The within-subject coefficient of variation did not exceed 2.1% for intraobserver and 2.8% for interobserver measurements, and reliability remained excellent when the acute and chronic phases were analyzed separately (all ICCs ≥ 0.96; Table 5).

In linear mixed-effects models adjusted for age, sex, and vascular comorbidities, all retinal layer, retinal vessel, and subfoveal choroidal measurements showed a significant decrease from the acute phase to the final visit in the affected eye (all *p* < 0.001). For peripapillary choroidal thickness, the adjusted models demonstrated a significant increase only in the inferior quadrant (β = +26.80 µm, 95% CI 10.55 to 43.05; *p* = 0.001), whereas the superior, temporal, and nasal quadrants did not reach significance (*p* = 0.056, 0.309, and 0.114, respectively; Table 6). The eye × visit interaction confirmed a significantly greater decrease in the affected eye than in the fellow eye for both subfoveal choroidal thickness (β = −32.43 µm; *p* < 0.001) and total macular thickness (β = −121.97 µm; *p* < 0.001), with no significant change in the fellow eye (Table 7).

**Figure 6:**
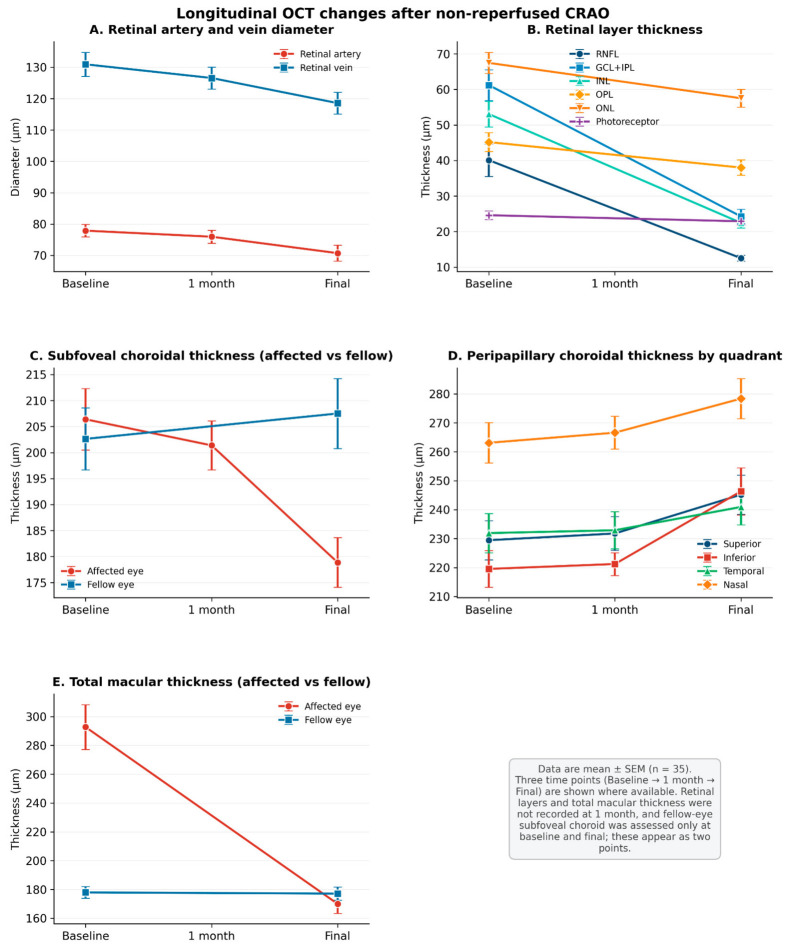
Longitudinal OCT changes after non-reperfused CRAO. (**A**) Mean retinal artery and retinal vein diameters at baseline, 1 month, and final follow-up. (**B**) Mean retinal layer thicknesses over time, including retinal nerve fiber layer (RNFL), ganglion cell layer plus inner plexiform layer (GCL + IPL), inner nuclear layer (INL), outer plexiform layer (OPL), outer nuclear layer (ONL), and photoreceptor layer. (**C**) Subfoveal choroidal thickness trajectories in affected and fellow eyes. (**D**) Peripapillary choroidal thickness over time by quadrant. (**E**) Total macular thickness trajectories in affected and fellow eyes. Data are presented as mean ± SEM. Three time points (baseline, 1 month, and final follow-up) are shown where available. Retinal layer thickness and total macular thickness were assessed at baseline and final follow-up, whereas fellow-eye subfoveal choroidal thickness was available at baseline and final follow-up only.

The longitudinal trajectories of retinal vascular, retinal layer, and choroidal parameters are summarized in Figure 6.

## 4. Discussion

In this study, we demonstrated distinct temporal changes in retinal morphology in eyes with central retinal artery occlusion, including progressive inner retinal atrophy, retinal vessel narrowing, and dynamic alterations in choroidal thickness across the acute and chronic phases. Using OCT-based quantitative analysis, our findings provide an integrated structural characterization of CRAO and highlight the heterogeneous involvement of retinal and choroidal compartments. The combined evaluation of retinal layer thickness, retinal vessel caliber, and regional choroidal thickness within the same persistently non-reperfused cohort represents a central contribution of this study and may provide objective reference data for future comparative or prognostic investigations. Notably, while most retinal parameters exhibited progressive thinning over time, peripapillary choroidal thickness showed a unique pattern, with relative preservation in the acute phase followed by a significant increase during follow-up in the absence of reperfusion [14].

Analysis of retinal vessel calibers revealed a delayed vascular response following CRAO. Retinal artery and vein diameters were comparable to those of healthy eyes during the acute phase; however, a clear narrowing of both arterioles and venules became evident in the chronic phase, suggesting progressive microvascular remodeling rather than an immediate post-occlusive change. In parallel, macular segmentation demonstrated that ischemic damage predominantly affected the inner retinal layers at the final follow-up. Significant thinning was observed in the retinal nerve fiber layer, ganglion cell layer, inner plexiform layer, and inner nuclear layer, reflecting their primary dependence on the central retinal artery. The relatively high outer plexiform layer (OPL) thickness values observed in the acute phase are likely attributable to ischemia-related edema and OCT segmentation characteristics, rather than intrinsic thickening of the OPL itself.

Among all segmented layers, the retinal nerve fiber layer exhibited the most dynamic structural response, characterized by prominent early thickening followed by marked thinning over time, likely representing acute ischemic edema and subsequent axonal loss. In contrast, the outer retinal layers—particularly the photoreceptor layer—showed minimal thinning, underscoring a gradient of ischemic vulnerability with decreasing structural involvement as the distance from the central retinal artery–supplied region increased.

Choroidal thickness analysis demonstrated divergent temporal patterns, with a gradual decrease in subfoveal choroidal thickness over time, whereas peripapillary choroidal thickness showed a progressive increase during follow-up. These contrasting changes further support the concept of heterogeneous choroidal involvement in CRAO. In the present study, the primary driver of the observed retinal, vascular, and choroidal alterations was the persistence of central retinal artery occlusion. Although recanalization was observed in a limited number of patients undergoing hyperbaric oxygen therapy due to distal migration of the occluding thrombus, these cases were excluded in order to specifically evaluate the structural consequences of sustained arterial occlusion.

Visual outcomes in CRAO are known to be profoundly poor, reflecting the severity of ischemic injury. In our cohort, visual acuity was limited to hand movements or finger counting in most patients, consistent with previous reports. MacGrory et al. described retinal artery occlusion as the “drooping” of the eye and reported that 74% of patients had visual acuity at the level of finger counting or worse, supporting the severe visual impairment observed in our study [15].

Quantitative evaluation of retinal vessel diameters in CRAO remains limited in the literature, despite frequent qualitative descriptions of vascular narrowing. While vessel attenuation is considered an inherent feature of the disease, few studies have provided objective measurements. Tripathy et al. demonstrated progressive narrowing of retinal arterioles following CRAO [16,17]. In our study, mean retinal arteriole and venule diameters during the acute phase remained within normal limits, suggesting that vascular caliber changes did not occur immediately after arterial occlusion [18]. In contrast, significant narrowing of both arterioles and venules was observed at the 6-month follow-up, indicating delayed microvascular remodeling as a component of chronic ischemic injury.

In a study by Tuncer et al., the mean subfoveal choroidal thickness (SFCT) in healthy Turkish individuals was reported as 265.86 ± 60.32 µm [19]. In our cohort, SFCT measured 206.40 ± 34.91 µm during the acute phase, which was comparable to values reported in normal populations. However, a significant reduction in SFCT was observed at the final follow-up, with mean thickness decreasing to 178.86 ± 28.28 µm (*p* = 0.009), indicating progressive subfoveal choroidal thinning over time. Similar chronic-phase reductions in SFCT among patients with central retinal artery occlusion have also been reported by Ahn et al. [10].

Detailed macular segmentation studies in retinal artery occlusion remain limited. In the present study, automated macular segmentation using spectral-domain OCT enabled a comprehensive assessment of layer-specific structural changes. Consistent with the ischemic nature of CRAO, the most pronounced alterations were observed in the inner retinal layers across all patients [20]. Falkenberry et al. similarly reported inner retinal thickening during the acute phase followed by thinning in the chronic phase, supporting our observations [21]. Our study further extends these findings by quantitatively demonstrating the temporal evolution of these changes between the acute and chronic stages.

Data on peripapillary choroidal thickness in CRAO are scarce, and quadrant-based evaluations are particularly limited. Given the known regional variability of PCT, measurements were obtained from the superior, inferior, nasal, and temporal quadrants in our study [22]. Shibata et al. reported the inferior quadrant to be thinner than other regions in healthy eyes [23], underscoring the importance of quadrant-specific assessment. Our findings build on this concept by demonstrating dynamic, quadrant-dependent changes in peripapillary choroidal thickness during the course of CRAO.

Erbağcı et al. reported mean peripapillary choroidal thickness values in healthy eyes as 225 ± 57 µm in the superior quadrant, 183 ± 47 µm in the inferior quadrant, 220 ± 57 µm in the temporal quadrant, and 233 ± 59 µm in the nasal quadrant [24]. In our acute-phase measurements, PCT values were comparable to those of healthy controls, with the inferior quadrant remaining relatively thinner than the other regions (superior 229.46 ± 40.13 µm; inferior 219.54 ± 37.59 µm; temporal 231.91 ± 40.09 µm; nasal 263.11 ± 41.13 µm). These findings suggest that peripapillary choroidal thickness is unlikely to represent a predisposing risk factor in CRAO, and that the observed changes are more likely secondary to disease-related hemodynamic alterations.

The increase in peripapillary choroidal thickness should nonetheless be interpreted with caution. Although unadjusted paired comparisons indicated an increase across all quadrants, in covariate-adjusted linear mixed-effects models this increase reached statistical significance only in the inferior quadrant (β = +26.80 µm, *p* = 0.001), whereas the superior, temporal, and nasal quadrants did not (*p* = 0.056–0.309). Moreover, the distributions of peripapillary choroidal thickness differences deviated from normality, so these parametric estimates should be considered together with the corresponding non-parametric analyses. Accordingly, the apparent peripapillary choroidal thickening—particularly outside the inferior quadrant—may partly reflect measurement variability and the limited sample size and should be confirmed in larger, prospective cohorts.

In contrast, increased PCT has been reported in anterior ischemic optic neuropathy (AION) and has been proposed as a potential risk factor in that condition (Fard et al.) [25,26], highlighting important pathophysiological differences between these two ischemic entities despite superficial clinical similarities.

The present study was not designed to evaluate the therapeutic efficacy of hyperbaric oxygen therapy. By restricting the cohort to non-reperfused CRAO cases, our primary objective was to isolate and characterize the natural structural evolution associated with persistent retinal ischemia. Accordingly, the absence of significant morphological differences between patients who received hyperbaric oxygen therapy and those who did not should not be interpreted as evidence against the efficacy of this treatment modality.

At the final follow-up, a significant increase in peripapillary choroidal thickness was observed across all quadrants (superior 245.20 ± 39.99 µm, inferior 246.34 ± 48.24 µm, temporal 240.94 ± 36.33 µm, nasal 278.37 ± 41.15 µm; all *p* < 0.05). This finding suggests that peripapillary choroidal thickening represents an active and dynamic response to persistent retinal ischemia rather than a static structural change. When considered together with the concurrent reduction in subfoveal choroidal thickness, this pattern suggests region-specific rather than uniform choroidal remodeling after CRAO. Several mechanisms may contribute to this phenomenon, including chronic venous congestion distal to the occlusion, compensatory dilation of the choroidal vasculature, and increased recruitment of collateral circulation through choroidal–retinal anastomotic pathways.

Experimental and clinical studies have suggested that, in the setting of sustained arterial occlusion, the choroid may act as a secondary vascular reservoir, partially compensating for reduced retinal perfusion. Retinal vascular occlusive events are known to disrupt normal ocular hemodynamics and may lead to secondary venous congestion distal to the site of occlusion, resulting in vascular engorgement and altered blood flow dynamics, which have been shown to influence choroidal structure and thickness in retinal vascular occlusive diseases. The peripapillary region, in particular, may be predisposed to such adaptive changes due to its rich vascular supply and anatomical proximity to the optic nerve head. In this context, the progressive increase in peripapillary choroidal thickness observed in our study may reflect a compensatory hemodynamic adaptation aimed at maintaining residual tissue perfusion in the absence of recanalization [27,28,29].

In addition to hemodynamic adaptations, retinal ischemia may induce upregulation of vascular endothelial growth factor (VEGF), increasing vascular permeability and potentially contributing to peripapillary choroidal thickening in the setting of sustained arterial occlusion [30,31].

Although the clinical implications of peripapillary choroidal thickening are not yet fully understood, our findings raise the possibility that enhancement of collateral blood flow could represent a potential therapeutic target in CRAO. Further longitudinal and interventional studies are warranted to clarify whether modulation of choroidal perfusion may influence structural outcomes or visual prognosis in affected patients.

One important limitation of this study is the relatively small sample size, which may have reduced statistical power, particularly for subgroup analyses such as hyperbaric oxygen therapy; therefore, non-significant findings should be interpreted with caution. Although the hyperbaric oxygen therapy protocol applied in our study was consistent with the recommended treatment algorithm, several factors limited a clear evaluation of its effects on visual and structural outcomes. Some patients were unable to complete the planned treatment sessions due to elevations in systemic arterial blood pressure during therapy, while others missed sessions because of advanced age and limited tolerance, resulting in reduced treatment adherence. Moreover, as arterial occlusion persisted in the analyzed cohort, morphological changes were largely similar between treated and untreated eyes, further limiting a definitive assessment of the structural impact of hyperbaric oxygen therapy.

Although retinal and vascular measurements were performed manually by a single primary observer, intra- and interobserver reliability were formally assessed in a representative subset of eyes and were excellent for all manually measured parameters (Table 5), supporting the reproducibility of these measurements. Choroidal thickness has been shown to be influenced by diurnal variation, as reported by Tan et al. [32], as well as by ocular biometric factors such as axial length and refractive error, as demonstrated by Tuncer et al. [19]; however, these parameters were not evaluated in the present study and should therefore be considered limitations when interpreting choroidal thickness measurements.

Additional limitations include the retrospective design of the study. Moreover, because the cohort was restricted to persistently non-reperfused CRAO cases, the findings may not be generalizable to reperfused CRAO. Although automated OCT-based segmentation was employed, minor segmentation inaccuracies may occur in severely atrophic retinas, potentially influencing layer-specific thickness measurements. Furthermore, the relatively limited duration of follow-up precluded assessment of longer-term structural remodeling. Future prospective studies with larger cohorts, extended follow-up periods, and standardized imaging protocols are warranted to further elucidate the morphological evolution of central retinal artery occlusion and its clinical implications.

## 5. Conclusions

Central retinal artery occlusion is associated with pronounced and time-dependent structural alterations involving the retina, retinal vasculature, and choroid. During follow-up, progressive narrowing of retinal vessels and marked atrophic changes predominantly affecting the inner retinal layers were observed, whereas thinning of the outer retinal layers was relatively limited. Subfoveal choroidal thickness demonstrated significant thinning over time, while peripapillary choroidal thickness exhibited a distinct increase, highlighting a region-specific and heterogeneous choroidal response to persistent ischemia.

These findings underscore the value of comprehensive OCT-based structural assessment in CRAO, even in cases without early reperfusion, as morphological changes appeared comparable regardless of treatment status. The integrated quantitative evaluation of retinal layers, retinal vessel diameters, and choroidal thickness may provide objective reference data for future studies and clinically meaningful biomarkers for the diagnosis, monitoring, and longitudinal assessment of disease progression in patients with central retinal artery occlusion.

## Figures and Tables

**Table 1:** Retinal Artery and Vein Diameters According to HBOT Subgroups.

Parameter	Baseline (µm)	1-Month (µm)	Final Follow-Up (µm)	*p*-Value
HBOT (+)–retinal artery diameter	80.60 ± 9.85	76.77 ± 8.17	73.84 ± 14.14	0.186
HBOT (−)–retinal artery diameter	71.20 ± 13.94	70.98 ± 12.97	63.00 ± 15.40	>0.05
HBOT (+)–retinal vein diameter	135.92 ± 21.04	129.88 ± 20.48	121.24 ± 20.86	0.270
HBOT (−)–retinal vein diameter	118.50 ± 22.29	117.68 ± 22.67	111.90 ± 19.37	>0.05

Values are presented as mean ± standard deviation. Between-group comparisons were performed using the Mann–Whitney U test.

**Table 2:** Macular Segmentation and Comparison of Baseline and Final Follow-up Measurements in All Patients.

Retinal Layer	Baseline (µm)	Final Follow-Up (µm)	*p*-Value
RNFL	40.09 ± 26.86	12.51 ± 4.99	0.001
Ganglion cell + IPL	61.20 ± 25.83	24.26 ± 12.11	0.001
INL	53.06 ± 21.31	22.46 ± 8.50	0.001
OPL	45.17 ± 15.62	38.00 ± 12.86	0.05
ONL	67.46 ± 17.54	57.51 ± 15.00	0.05
Photoreceptor layer	24.60 ± 7.30	22.89 ± 5.98	0.20

Abbreviations: RNFL, retinal nerve fiber layer; IPL, inner plexiform layer; INL, inner nuclear layer; OPL, outer plexiform layer; ONL, outer nuclear layer.

**Table 3:** Comparison of Baseline and Final Follow-up Macular Segmentation Measurements According to Hyperbaric Oxygen Therapy.

Macular Segmentation	HBOT (+) (*n* = 25)	HBOT (−) (*n* = 10)	Between-Group *p* Value
RNFL (baseline)	37.08 ± 15.56	47.60 ± 44.64	0.957
RNFL (final follow-up)	12.32 ± 4.43	13.00 ± 6.43	0.999
RNFL baseline–final *p* value	0.001	0.001	
GCL + IPL (baseline)	61.28 ± 24.43	61.00 ± 30.48	0.956
GCL + IPL (final follow-up)	26.16 ± 13.60	19.50 ± 5.06	0.510
GCL + IPL baseline–final *p* value	0.001	0.001	
INL (baseline)	49.56 ± 17.26	61.80 ± 28.32	0.465
INL (final follow-up)	22.28 ± 9.45	22.90 ± 5.90	0.332
INL baseline–final *p* value	0.001	0.005	
OPL (baseline)	42.32 ± 14.20	52.30 ± 17.45	0.089
OPL (final follow-up)	36.24 ± 12.12	42.40 ± 14.26	0.265
OPL baseline–final *p* value	0.001	0.005	

Abbreviation: HBOT, hyperbaric oxygen therapy. Values are presented as mean ± standard deviation.

**Table 4:** Peripapillary Choroidal Thickness Measurements During Follow-up.

Quadrant	Baseline (µm)	1-Month (µm)	Final Follow-Up (µm)
Superior quadrant	229.46 ± 40.13	231.78 ± 34.57	245.20 ± 39.99
Inferior quadrant	219.54 ± 37.59	221.23 ± 23.22	246.34 ± 48.24
Temporal quadrant	231.91 ± 40.09	232.89 ± 38.07	240.94 ± 36.33
Nasal quadrant	263.11 ± 41.13	266.61 ± 33.59	278.37 ± 41.15

Data are presented as mean ± standard deviation. Baseline and final follow-up comparisons were statistically significant for all quadrants. (superior *p* = 0.011, inferior *p* = 0.001, temporal *p* = 0.038, nasal *p* = 0.006).

**Table 5:** Intra- and interobserver reliability of manually measured parameters.

Parameter	Intraobserver ICC (95% CI)	Interobserver ICC (95% CI)	Intraobserver CV (%)	Interobserver CV (%)
Retinal artery diameter	0.98 (0.96–0.99)	0.97 (0.95–0.99)	2.08	2.78
Retinal vein diameter	1.00 (0.99–1.00)	0.99 (0.98–0.99)	1.34	1.94
Subfoveal choroidal thickness	1.00 (0.99–1.00)	0.99 (0.98–1.00)	0.84	1.26
Peripapillary choroid, superior	1.00 (0.99–1.00)	0.99 (0.99–1.00)	0.93	1.24
Peripapillary choroid, inferior	1.00 (0.99–1.00)	1.00 (0.99–1.00)	1.15	1.36
Peripapillary choroid, temporal	1.00 (0.99–1.00)	0.99 (0.99–1.00)	0.87	1.09
Peripapillary choroid, nasal	1.00 (0.99–1.00)	0.99 (0.99–1.00)	0.79	1.02

Data are based on a randomly selected subset of 20 eyes, with acute- and chronic-phase measurements pooled. ICCs were calculated using a two-way random-effects model with absolute agreement (single measures); values > 0.90 indicate excellent reliability. CI, confidence interval; CV, coefficient of variation; ICC, intraclass correlation coefficient.

**Table 6:** Adjusted linear mixed-effects models for baseline-to-final longitudinal change in the affected eye.

Outcome	β (µm)	95% CI	*p* Value
Retinal artery diameter	−7.17	−10.08 to −4.26	<0.001
Retinal vein diameter	−12.37	−18.07 to −6.67	<0.001
RNFL thickness	−27.57	−35.56 to −19.58	<0.001
GCL + IPL thickness	−36.94	−44.26 to −29.62	<0.001
INL thickness	−30.60	−36.69 to −24.51	<0.001
OPL thickness	−7.17	−9.15 to −5.20	<0.001
ONL thickness	−9.94	−12.98 to −6.90	<0.001
Photoreceptor layer thickness	−1.71	−2.63 to −0.80	<0.001
Subfoveal choroidal thickness	−27.54	−34.84 to −20.25	<0.001
PCT superior	15.74	−0.37 to 31.86	0.056
PCT inferior	26.80	10.55 to 43.05	0.001
PCT temporal	9.03	−8.38 to 26.43	0.309
PCT nasal	15.26	−3.66 to 34.18	0.114
Total macular thickness	−122.77	−144.04 to −101.50	<0.001

β represents the adjusted mean change from baseline (acute phase) to final follow-up; negative values indicate a decrease. All models included patient identity as a random intercept and were adjusted for age, sex, diabetes mellitus, hypertension, and cardiovascular disease. Axial length and refractive error were not available and were therefore not included. Peripapillary choroidal thickness (PCT) difference distributions deviated from normality (Shapiro–Wilk *p* < 0.01); these parametric estimates are reported for completeness and should be interpreted together with the corresponding non-parametric analysis. CI, confidence interval; GCL + IPL, ganglion cell–inner plexiform layer; INL, inner nuclear layer; ONL, outer nuclear layer; OPL, outer plexiform layer; PCT, peripapillary choroidal thickness; RNFL, retinal nerve fiber layer.

**Table 7:** Affected eye versus fellow eye: linear mixed-effects models with an eye × visit interaction.

Outcome	Affected Eye, Baseline → Final (µm)	Fellow Eye, Baseline → Final (µm)	Eye × Visit β (95% CI), µm	*p* Value
Subfoveal choroidal thickness	206.40 ± 34.91 → 178.86 ± 28.28	202.63 ± 35.23 → 207.51 ± 39.83	−32.43 (−44.70 to −20.16)	<0.001
Total macular thickness	292.74 ± 92.03 → 169.97 ± 39.24	177.91 ± 24.14 → 177.11 ± 26.83	−121.97 (−151.22 to −92.72)	<0.001

Descriptive values are mean ± SD. The eye × visit interaction tests whether the change over time differed between the affected and fellow eyes; a negative β indicates a significantly greater decrease in the affected eye relative to the fellow eye. Both models included patient identity as a random intercept and were adjusted for age, sex, diabetes mellitus, hypertension, and cardiovascular disease. The fellow eye showed no significant change over time (subfoveal choroidal thickness, *p* = 0.27; total macular thickness, *p* = 0.94), and the two eyes did not differ at baseline for subfoveal choroidal thickness (*p* = 0.39). CI, confidence interval; SD, standard deviation.

## Data Availability

The datasets generated and/or analyzed during the current study are not publicly available due to patient privacy and institutional restrictions but are available from the corresponding author upon reasonable request.
